# Zinc Chloride Transiently Maintains Mouse Embryonic Stem Cell Pluripotency by Activating Stat3 Signaling

**DOI:** 10.1371/journal.pone.0148994

**Published:** 2016-02-24

**Authors:** Jing Hu, Zhiyong Yang, Jinbo Wang, Jia Yu, Jing Guo, Shiying Liu, Chunmei Qian, Liwen Song, Yi Wu, Jiajing Cheng

**Affiliations:** 1 Department of Obstetrics and Gynecology, Shanghai Tenth People's Hospital, Tongji University School of Medicine, Shanghai, China; 2 The First Clinical Medical College of Nanjing Medical University, Nanjing, Jiangsu, China; Universidade de São Paulo, BRAZIL

## Abstract

An improved understanding of the pluripotency maintenance of embryonic stem (ES) cells is important for investigations of early embryo development and for cell replacement therapy, but the mechanism behind pluripotency is still incompletely understood. Recent findings show that zinc, an essential trace element in humans, is critically involved in regulating various signaling pathways and genes expression. However, its role in ES cell fate determination remains to be further explored. Here we showed that 2μM zinc chloride (ZnCl_2_) transiently maintained mouse ES cell pluripotency *in vitro*. The cultured mouse ES cells remained undifferentiated under 2μM ZnCl_2_ treatment in leukemia inhibitory factor (LIF) withdrawal, retinoic acid (RA) or embryoid bodies (EBs) differentiation assays. In addition, ZnCl_2_ increased pluripotency genes expression and inhibited differentiation genes expression. Further mechanistic studies revealed that ZnCl_2_ transiently activated signal transducers and activators of transcription 3 (Stat3) signaling through promoting Stat3 phosphorylation. Inhibition of Stat3 signaling abrogated the effects of ZnCl_2_ on mouse ES cell pluripotency. Taken together, this study demonstrated a critical role of zinc in the pluripotency maintenance of mouse ES cells, as well as an important regulator of Stat3 signaling.

## Introduction

ES cells are derived from early preimplantation embryos and they have two remarkable properties: the ability to differentiate into virtually all types of cells of the adult body, known as pluripotency; and the capacity for unlimited proliferation while maintaining the pluripotent state, known as self-renewal [1]. These characteristics render ES cells a useful and available tool for investigating the molecular and cellular control of embryogenesis and the mechanisms regarding embryonic developmental diseases [2–5].

Natural or synthetic small molecule compounds are important tools for controlling and manipulating ES cell fate, state, or function *in vitro* and *in vivo* [6–8]. 6-bromoindirubin-3’-oxime, an inhibitor of glycogen synthase kinase-3 (Gsk3), also an activator of Wnt pathway, is the first pharmacological agent shown to maintain ES cell pluripotency and self-renewal [9]. Small molecules can replace LIF and serum/BMP to maintain self-renewal and pluripotency of ES cells through regulating various signaling pathways. In a combination of three selective small-molecule inhibitors (CHIR99021, SU5402 and PD184352), which target Gsk3, fibroblast growth factor receptor tyrosine kinases, and mitogen-activated protein kinase kinase (Mek), respectively, mouse ES cells maintained an undifferentiated state and a faster self-renewal rate comparable to that in LIF plus serum/BMP medium [10]. In this new field, more and more novel small molecules functioned in ES cell fate regulation have been identified in recent years. For example, a recent breakthrough demonstrated that mouse pluripotent stem cells could be induced from somatic cells through using specific small molecule compounds without the ectopic expression of the well-known Yamanaka factors OKSM (Oct4, klf4, Sox2 and c-Myc) [11]. Importantly, compared with genetic manipulation, these small molecules provide researchers more controllable and reversible approaches for ES cell fate regulation in regenerative medicine.

Mouse ES cells can be maintained in undifferentiated state in culture medium with the presence of LIF [12, 13]. LIF stimulation leads to the phosphorylation of Stat3, which is important for the pluripotency maintenance of mouse ES cells [14, 15]. LIF/Stat3 signaling pathway plays a central role in the maintenance of the pluripotency of mouse ES cells *in vitro*. Although the mechanisms regarding genetic or epigenetic regulation of Stat3 pathway in ES cells have been extensively studied [16–20], the regulation of Stat3 pathway by small molecules in maintaining pluripotency remains less understood.

The complex cellular environment contains many nature small molecules, such as inorganic, organic salts and amino acids. These small molecules may participate critically in the regulation of early embryo development. Zinc, an essential trace element in humans, is an integral component of many proteins, such as metalloenzymes and zinc finger transcription factors. The regulatory roles of zinc in many biological processes including proliferation, growth, development and metabolism have been broadly explored. For example, zinc is very important for cell proliferation, especially for DNA synthesis and mitosis [21]. Zinc also regulates the metabolism of cGMP and the activities of protein kinase C [21]. In addition, some recent studies showed that zinc participated in regulating gene expression through metalresponsive transcription factor 1 [22, 23]. Recently, there is increasing evidences demonstrating that zinc has been identified as an intracellular second messenger involved in regulating various signaling pathways [24–26].

Many studies reveal that zinc is important for ES cell biology. For example, Klf4, a zinc finger transcription factor, is an essential member of the core pluripotency transcriptional network in ES cells [27–29]. A recent study demonstrated that zinc was required for embryonic development, and supplementation with adequate zinc improved the viability of porcine embryos *in vitro* [30]. Commercially available culture media for mouse ES cells do not contain any zinc ion. Therefore, little information exists regarding the effect of zinc on mouse ES cells *in vitro*, indicating that these media are ideal tools for investigating the function of zinc in mouse ES cells. A study reported that zinc chloride (ZnCl_2_) stimulated mouse ES cell proliferation through PI3K/Akt, MAPKs, and mTOR signaling pathways [31]. However, the effect of zinc on ES cell fate determination remains poorly understood. ZnCl_2_ is a natural small molecule, which is an important component of extracellular and intracellular environment. Understanding of the mechanisms by which ZnCl_2_ regulates mouse ES cell fate determination will be helpful to advance our knowledge about extrinsic control of pluripotent stem cells. In this study, we designed to investigate the effects of ZnCl_2_ on mouse ES cell pluripotency and differentiation as well as the related regulatory mechanisms.

## Materials and Methods

### Mouse ES cell culture and treatment

The mouse ES cell line ES-E14TG2a, purchased from the American Type Culture Collection (ATCC CRL-1821), was cultured on cell culture plates coated with 0.1% gelatin (Millipore, Billerica, MA) and in high-glucose Dulbecco’s modified Eagle’s medium (Gibco, Grand Island, NY) supplemented with 15% (v/v) ES cell qualified fetal bovine serum (Gibco), 1% (v/v) penicillin and streptomycin (Gibco), 0.1mM β-mercaptoethanol (Sigma-Aldrich, St. Louis, MO), 0.1mM nonessential amino acids (Gibco), 2mM L-glutamine (Gibco), and 1,000units/ml LIF (Millipore).

ZnCl_2_ (AMRESCO, Solon, OH) at different final concentrations of 0.02μM, 0.2μM, 2μM or 20μM were added into medium, and mouse ES cells were incubated with ZnCl_2_ for different time in different experiments.

For LIF withdrawal differentiation assay, mouse ES cells were cultured in medium containing all other supplements at the concentrations indicated above without LIF.

For RA differentiation assay, mouse ES cells were cultured in 6-well plates. 10μM Retinoic acid (Sigma-Aldrich) and ZnCl_2_ at different final concentrations were added into LIF withdrawal medium. After 48 hours, the cells were fixed for alkaline phosphatase staining or RNAs and proteins were collected for further analyses.

For EB differentiation assay, mouse ES cells were treated with 0.05%Trypsin/EDTA and then cultured in LIF withdrawal medium in Ultra Low Culture Dish (Corning, Corning, NY). ZnCl_2_ at different final concentrations were added into medium. Then, on day 6, RNAs and proteins were collected for further analyses.

The mouse ES cells were cultured in LIF withdrawal medium supplemented with Mek inhibitor PD0325901 (1μM) (Sigma-Aldrich) and Gsk3 inhibitor CHIR99021 (3μM) (Sigma-Aldrich) to sustain efficient self-renewal and pluripotency, known as 2i [10]. This was used as positive control in our study.

For AG490 (a Stat3 pathway inhibitor) inhibition experiment, AG490 (Sigma-Aldrich) at a final concentration of 30μM was added into LIF withdrawal medium. We treated mouse ES cells with AG490 and ZnCl_2_ simultaneously.

### Alkaline phosphatase staining

Cells were washed twice with PBS and fixed with 4% paraformaldehyde (Sigma-Aldrich) for approximately 1 min at room temperature. Then, cells were incubated with an alkaline phosphatase substrate solution (Sigma-Aldrich) for 10–15 minutes at room temperature. After a wash with PBS, the cells were photographed.

### Reverse Transcription, qRT-PCR

Total RNA was isolated using the Trizol reagent (Invitrogen, Carlsbad, CA) following the manufacturer’s instructions. For each sample, 500ng of RNA was reverse transcribed to cDNA using the Prime-Script RT reagent kit (TaKaRa, Dalian, China). The obtained cDNA was amplified with the Takara Ex Taq PCR kit (TaKaRa), and qRT-PCR amplification was conducted using the Stratagene Mx3000 QPCR system (Stratagene, Foster City, CA) and analyzed via the ΔΔCT method. The primer sequences used in these assays are available in S1 Table.

### Western Blotting

Cultured cells were lysed in strong RIPA buffer containing Halt Protease Inhibitor Cocktails (Thermo, Waltham, MA). The obtained protein concentrations were measured using a BCA protein assay kit (Pierce, Rockford, IL). Primary antibodies targeting OCT4 (Santa Cruz Biotechnology, Santa Cruz, CA), NANOG (Abcam, Cambridge, U.K), SOX2 (Abcam), STAT3 (Cell Signaling Technology, Boston, MA), STAT3 (pTyr705) (Cell Signaling Technology) and GAPDH (Santa Cruz Biotechnology) were incubated with the proteins overnight at 4℃, followed by incubation with the appropriate HRP (horseradish peroxidase) conjugated secondary antibodies. Detection of HRP was performed using the Super Signal West Pico Chemiluminescent Substrate (Pierce).

### Immunofluorescence staining

Cells cultured in 24-well dishes were fixed in 4% paraformaldehyde and permeabilized with 0.25% Triton X-100, followed by blocking with 10% FBS in PBS. Then cells were probed with primary antibody in 10% FBS overnight at 4°C and secondary antibody in 10% FBS for 2 hours at room temperature. Then nuclei were counterstained with Hoechst 33342 for 2 minutes. Cells were washed with and imaged in PBS. Primary antibodies used were OCT4 (Santa Cruz Biotechnology), NANOG (Abcam) and Ki67 (Abcam).

### Cell Counting Kit-8 (CCK8) cell proliferation assay

The cells were seeded in 96-well plates at a density of 1×10^3^ cells/well. After different treatment, the reagent (Dojindo, Kumamoto, Japan) was thawed for approximately 10 minutes in a water bath at 37°C, and 10 μl of the reagent was added to each well of the 96-well plate. Then the plate was incubated at 37°C for 1–4 hours. Finally, the absorbance at 450 nm was recorded with SpectraMax M5.

### Statistical Analysis

The error bars represent the SEM of three independent experiments. Data are represented as mean±SEM; n = 3. *, ** and *** indicate p<0.05, p<0.01, and p<0.001, respectively (Student’s t test).

## Results

### Zinc promotes mouse ES cell pluripotency in LIF withdrawal differentiation assay

**Fig 1:**
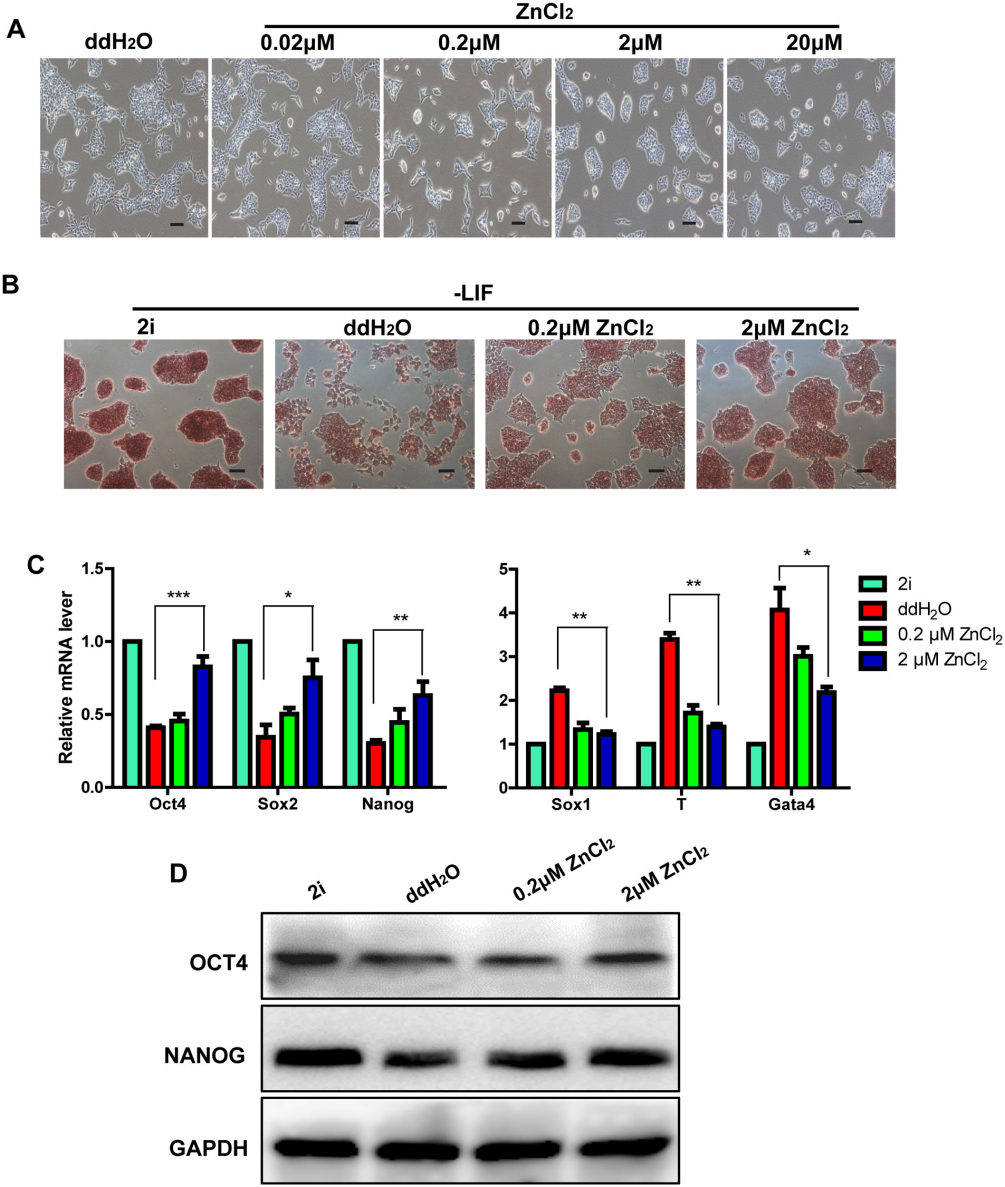
Zinc promotes mouse ES cell pluripotency in LIF withdrawal differentiation assay. All cells were cultured in LIF withdrawal medium. (A): Morphological differences between cells treated with ddH_2_O and ZnCl_2_ at the indicated concentrations for 48 hours. Bars = 40μm. (B): Alkaline phosphatase staining of cells treated with 2i, ddH_2_O and ZnCl_2_ (0.2μM and 2μM) for 48 hours. Bars = 40μm. (C): qRT-PCR analyses of the mRNA levels of pluripotency transcription factors (Oct4, Sox2 and Nanog) and lineage-specific markers (Sox1, T and Gata4) in cells treated with 2i, ddH_2_O and ZnCl_2_ (0.2μM and 2μM) for 48 hours. The data are displayed relative to the results of the 2i treatment group and represented as mean±SEM; n = 3. *, ** and *** indicate p<0.05, p<0.01, and p<0.001, respectively. (D): Western blot analyses of the protein levels of OCT4 and NANOG in cells treated 2i, ddH_2_O and with ZnCl_2_ (0.2μM and 2μM) for 48 hours.

In the current *in vitro* culture system, mouse ES cells require LIF to maintain their pluripotent state [12, 13]. To explore whether zinc supports the pluripotency maintenance of mouse ES cells, we incubated the cells with ZnCl_2_ at different concentrations (0.02μM, 0.2μM, 2μM, and 20μM) for 48 hours in LIF withdrawal medium. We then examined the morphology of treated cells. Compared with the negative control (ddH2O) and low concentration (0.02μM and 0.2μM) group, high concentration ZnCl_2_ (2μM and 20μM) maintained the clone morphology of ES cells and markedly reduced their spontaneous differentiation (Fig 1A). When the concentration increased, the clone morphology was more obvious. However, when the concentration reached 20μM, the clone morphology was not considerably improved compared to 2μM ZnCl_2_ treatment, indicating that the concentration of ZnCl_2_ reached saturation. Therefore, we chose 2μM as experimental concentration for the following experiments. Ying et al. reported that two potent selective small molecule inhibitors, PD0325901 and CHIR99021, which target Mek and Gsk3, respectively, are sufficient to sustain efficient mouse ES cell self-renewal and pluripotency [10]. Therefore, in our following experiments, we used these two molecules, known as 2i, as positive control. 2μM ZnCl_2_ treated cells had similar AP enzyme activity compared to 2i treated cells (Fig 1B). However, compared with ddH2O and 0.2μM ZnCl_2_ treatment, 2μM ZnCl_2_ treatment led to a stronger alkaline phosphatase (AP) enzyme activity, an indicative of pluripotency for mouse ES cells (Fig 1B). qRT-PCR and western-blot analyses revealed that 2μM ZnCl_2_ treatment significantly increased the expression levels of pluripotency markers, including Oct4, Sox2 and Nanog, which are essential for the pluripotency maintenance of mouse ES cells (Fig 1C and 1D). Moreover, 2μM ZnCl_2_ treatment also inhibited the mRNA levels of genes related to differentiation into the three embryonic germ layers, including Sox1, T and Gata4 (Fig 1C). Therefore, our results suggested that ZnCl_2_ at certain concentration promoted mouse ES cell pluripotency in LIF withdrawal medium.

### Zinc promotes mouse ES cell pluripotency in RA and EB differentiation assays

**Fig 2:**
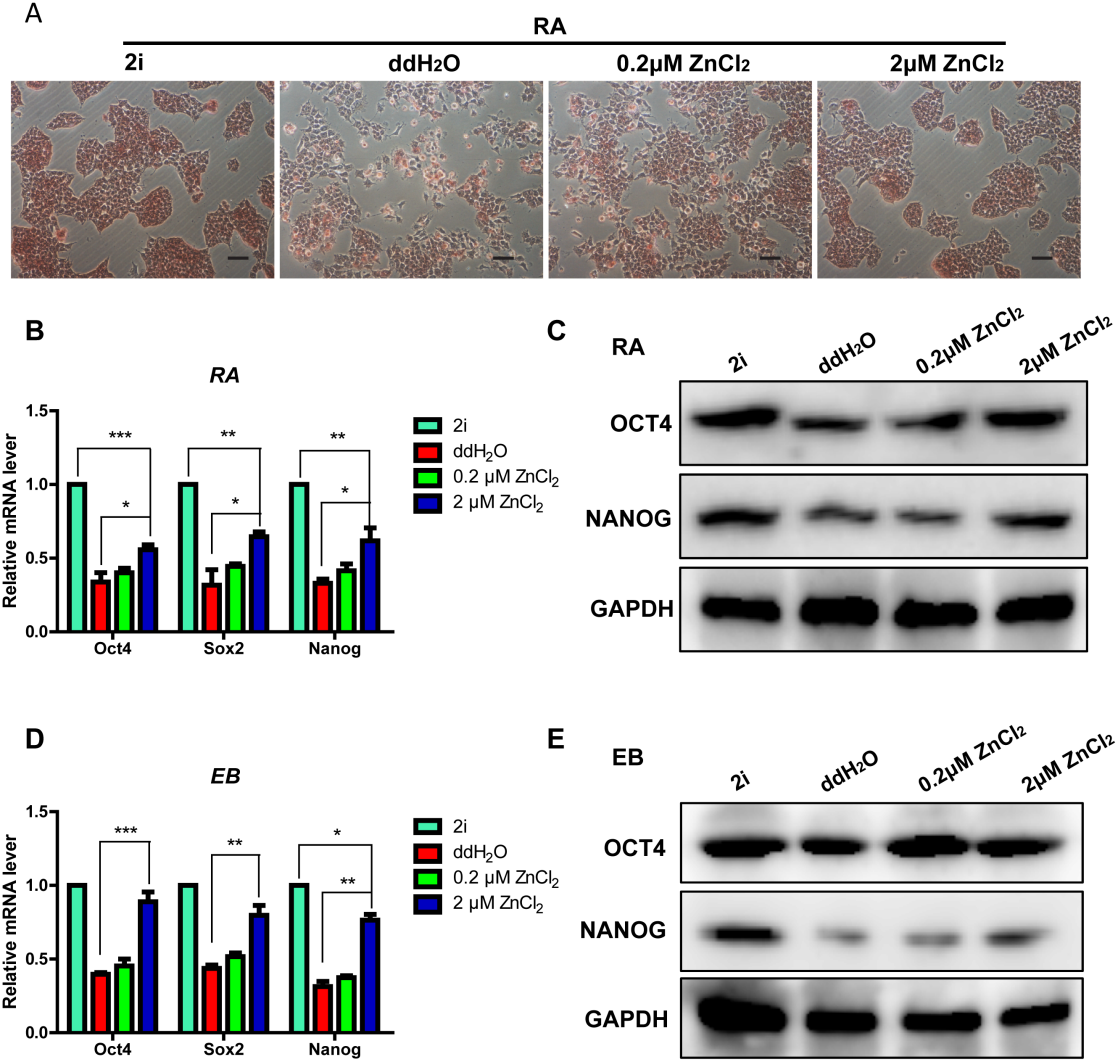
Zinc promotes mouse ES cell pluripotency in RA and EB differentiation assays. RA differentiation assay (A)-(C): all cells were treated with RA (10μM) for 48 hours and cultured in LIF withdrawal medium. (A) Alkaline phosphatase staining of cells treated with 2i, ddH_2_O and ZnCl_2_ (0.2μM and 2μM) for 48 hours. (B) qRT-PCR analyses of the mRNA levels of pluripotency transcription factors (Oct4, Sox2 and Nanog) in cells treated with 2i, ddH_2_O and ZnCl_2_ (0.2μM and 2μM) for 48 hours. The data are displayed relative to the results of the 2i treatment group and represented as mean±SEM; n = 3. *, ** and *** indicate p<0.05, p<0.01, and p<0.001, respectively. (C) Western blot analyses of the protein levels of OCT4 and NANOG in cells treated with 2i, ddH_2_O and ZnCl_2_ (0.2μM and 2μM) for 48 hours. EB differentiation assay (D)-(E). (D) qRT-PCR analyses of the mRNA levels of pluripotency transcription factors (Oct4, Sox2 and Nanog) in cells treated with 2i, ddH_2_O and ZnCl_2_ (0.2μM and 2μM) for 6 days. The data are displayed relative to the results of the 2i treatment group and represented as mean±SEM; n = 3. *, ** and *** indicate p<0.05, p<0.01, and p<0.001, respectively. (E) Western blot analyses of the protein levels of OCT4 and NANOG in cells treated with 2i, ddH_2_O and ZnCl_2_ (0.2μM and 2μM) for 6 days.

To further investigate the relationship between zinc and pluripotency, the process of differentiation was perturbed by the presence of 10μM RA, which is closely involved in mouse ES cell differentiation [32]. After 48 hours of RA exposure in LIF withdrawal medium, 2μM ZnCl_2_ treated cells showed a stronger AP enzyme activity and maintained a better clone morphology compared to ddH_2_O treated cells (Fig 2A). Moreover, compared with ddH_2_O treatment, 2μM ZnCl_2_ treatment obviously increased the mRNA and protein levels of pluripotent transcription factors Oct4, Sox2 and Nanog in RA differentiation assay (Fig 2B and 2C). In addition, we further confirmed the effect of zinc on the pluripotency by EB differentiation assay. We induced 2i, ddH_2_O and ZnCl_2_ treated mouse ES cells to form EBs and then analyzed the expression levels of pluripotent transcription factors via qRT-PCR and Western blot on EB Day 6. We found that 2μM ZnCl_2_ treatment elevated the mRNA and protein levels of Oct4, Sox2 and Nanog in EB differentiation assay compared to ddH_2_O treatment (Fig 2D and 2E).

ZnCl_2_ can promote pluripotent transcription factor expression. However, we also observed that the mRNA levels of Oct4, Sox2 and Nanog were slightly lower in 2μM ZnCl_2_ treated cells than that in 2i treated cells in RA and EB differentiation assay (Fig 2B and 2D). This may indicate that the effect of ZnCl_2_ on pluripotency maintenance is slightly weaker compared to 2i.

Taken together, our data presented above revealed that ZnCl_2_ at appropriate concentration promoted mouse ES cell pluripotency in different differentiation conditions.

### Zinc transiently maintains mouse ES cell pluripotency *in vitro*

**Fig 3:**
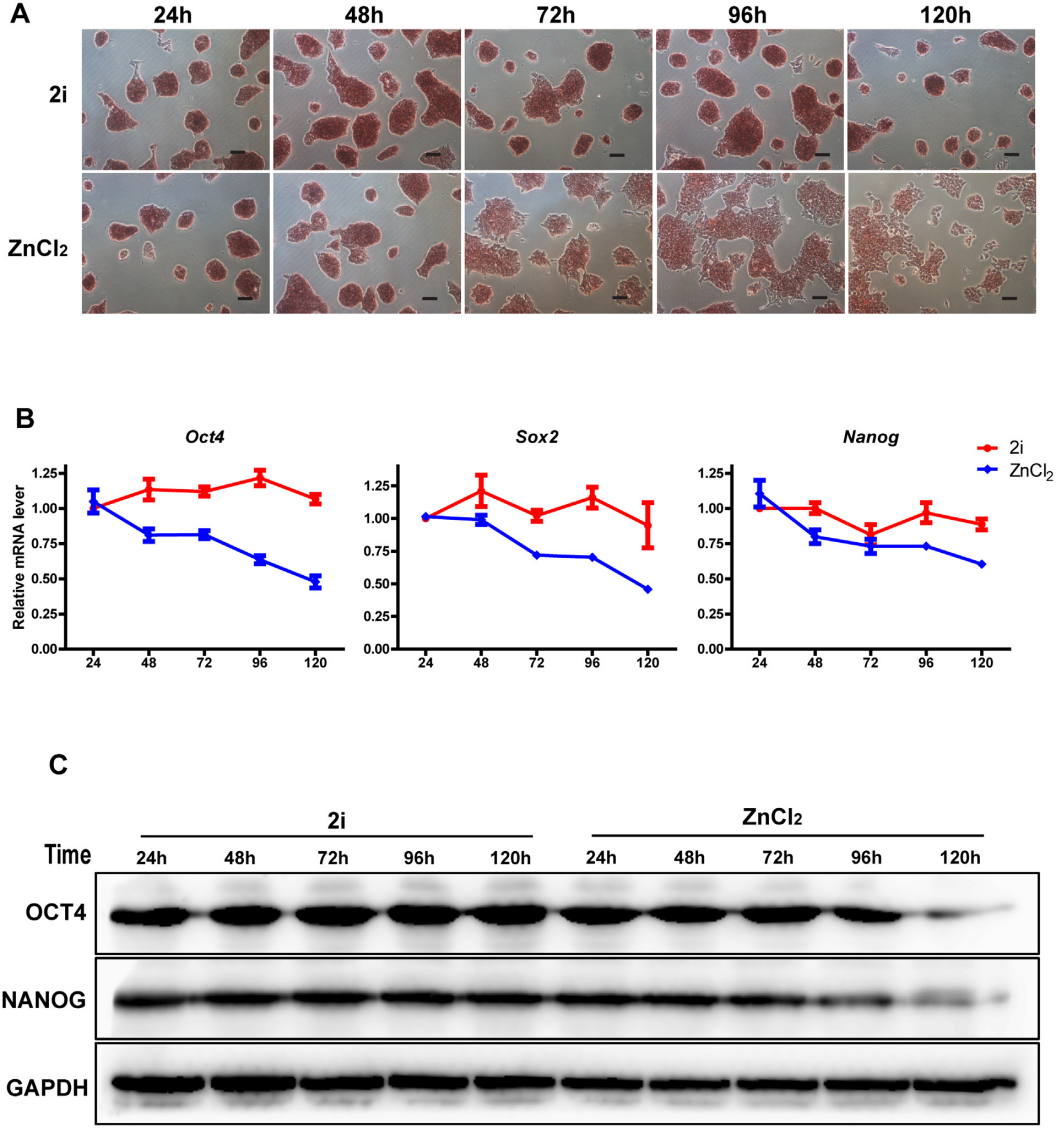
Zinc transiently maintains mouse ES cell pluripotency *in vitro*. All cells were cultured in LIF withdrawal medium. (A): Alkaline phosphatase staining of cells treated with 2i and 2μM ZnCl_2_ at the indicated time. Bars = 40μm. (B): qRT-PCR analyses of the mRNA levels of pluripotency transcription factors (Oct4, Sox2 and Nanog) in cells treated with 2i and 2μM ZnCl_2_ at the indicated time. The data are displayed relative to the results of the 2i treatment for 24 hours group and represented as mean±SEM; n = 3. (C): Western blot analyses of the protein levels of OCT4 and NANOG in cells treated with 2i and 2μM ZnCl_2_ at the indicated time.

Next, we tested whether 2μM ZnCl_2_ could maintain the pluripotency of mouse ES cells for more than 48 hours *in vitro*. Compared with 2i treated cells, 2μM ZnCl_2_ treated cells sustained similar clone morphology and AP enzyme activity after 24 hours or 48 hours incubation in LIF withdrawal medium (Fig 3A). However, as the cells were passaged and the incubation time was prolonged to 72 hours, 96 hours or 120 hours, 2μM ZnCl_2_ treated ES cells started the trend of differentiation, losing their clone morphology and AP enzyme activity gradually (Fig 3A). qRT-PCR and western-blot analyses also revealed that the expression levels of pluripotency markers decreased gradually after 2μM ZnCl_2_ treatment for 72 hours, 96 hours or 120 hours (Fig 3B and 3C). Thus, these data indicated that zinc transiently maintained mouse ES cell pluripotency *in vitro*.

### Zinc does not affect mouse ES cell pluripotency and proliferation in the presence of LIF

**Fig 4:**
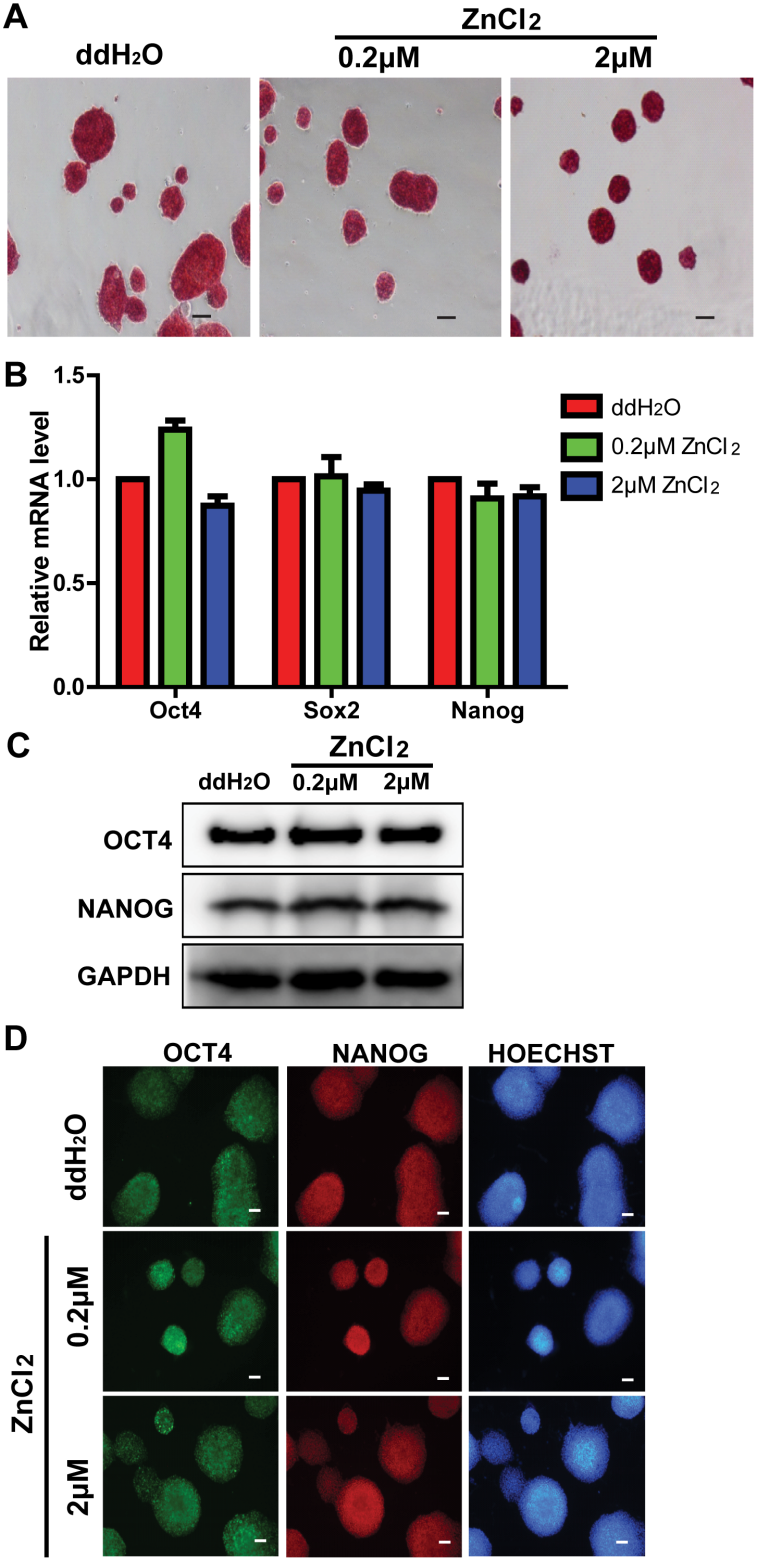
Zinc does not affect mouse ES cell pluripotency in the presence of LIF. All cells were cultured in medium supplemented with LIF. (A): Alkaline phosphatase staining of cells treated with ddH_2_O and ZnCl_2_ (0.2μM and 2μM) for 48 hours. Bars = 40μm. (B): qRT-PCR analyses of the mRNA levels of pluripotency transcription factors Oct4, Sox2 and Nanog in cells treated with ddH_2_O and ZnCl_2_ (0.2μM and 2μM) for 48 hours. The data are displayed relative to the results of the ddH_2_O treatment group and represented as mean±SEM; n = 3. (C): Western blot analyses of the protein levels of OCT4 and NANOG in cells treated with ddH_2_O and ZnCl_2_ (0.2μM and 2μM) for 48 hours. (D): Immunostaining images of cells treated with ddH_2_O and ZnCl_2_ (0.2μM and 2μM) for 48 hours. Cells were stained with antibodies against OCT4 and NANOG. Nuclei were counterstained with Hoechst33342. Bars = 40μm.

To investigate whether zinc has an impact on ES cell pluripotency when LIF is supplemented in the medium, we cultured the cells with ZnCl_2_ at different concentrations (0.2μM and 2μM) for 48 hours in medium supplemented with LIF and further detected the expression levels of pluripotency markers. After 48 hours of ZnCl_2_ exposure, ES cells maintained similar alkaline phosphatase enzyme activity (Fig 4A). The mRNA levels of Oct4, Sox2, and Nanog were without significant differences as well (Fig 4B). The protein levels of OCT4 and NANOG also maintained at similar levels as reflected by western blotting and immunocytochemistry analyses (Fig 4C and 4D). Interestingly, a previous study also showed that mouse ES cells remained undifferentiated under 72 hours of 40μM ZnCl_2_ exposure in LIF containing medium [31]. Therefore, our data indicated that 2μM ZnCl_2_ did not affect the pluripotency of mouse ES cells in the presence of LIF.

Meanwhile, we also tested whether zinc had an impact on ES cell proliferation when LIF was supplemented in the medium. Ki67 protein is a cellular marker for proliferation, which is present during all active phases of the cell cycle, but is absent from resting cells. After 48 hours of 2μM ZnCl_2_ treatment, ES cells exhibited similar Ki67 expression level as reflected by immunocytochemistry analysis (S1A Fig). ES cells also sustained similar growth rate through growth curves that were constructed using the CCK-8 assay (S1B Fig). These results indicated that 2μM ZnCl_2_ did not affect the proliferation of mouse ES cells. A previous study demonstrated that at appropriate concentrations (≥4μM), ZnCl_2_ increased cell cycle regulatory protein levels, [3H]-thymidine incorporation, and total cell numbers, but higher doses of ZnCl_2_ (≥200μM) blocked the proliferation-promoting effect [31]. This study, together with our work, suggests that ZnCl_2_ at different concentrations have different effects on ES cell proliferation.

### Zinc transiently activates Stat3 signaling

**Fig 5:**
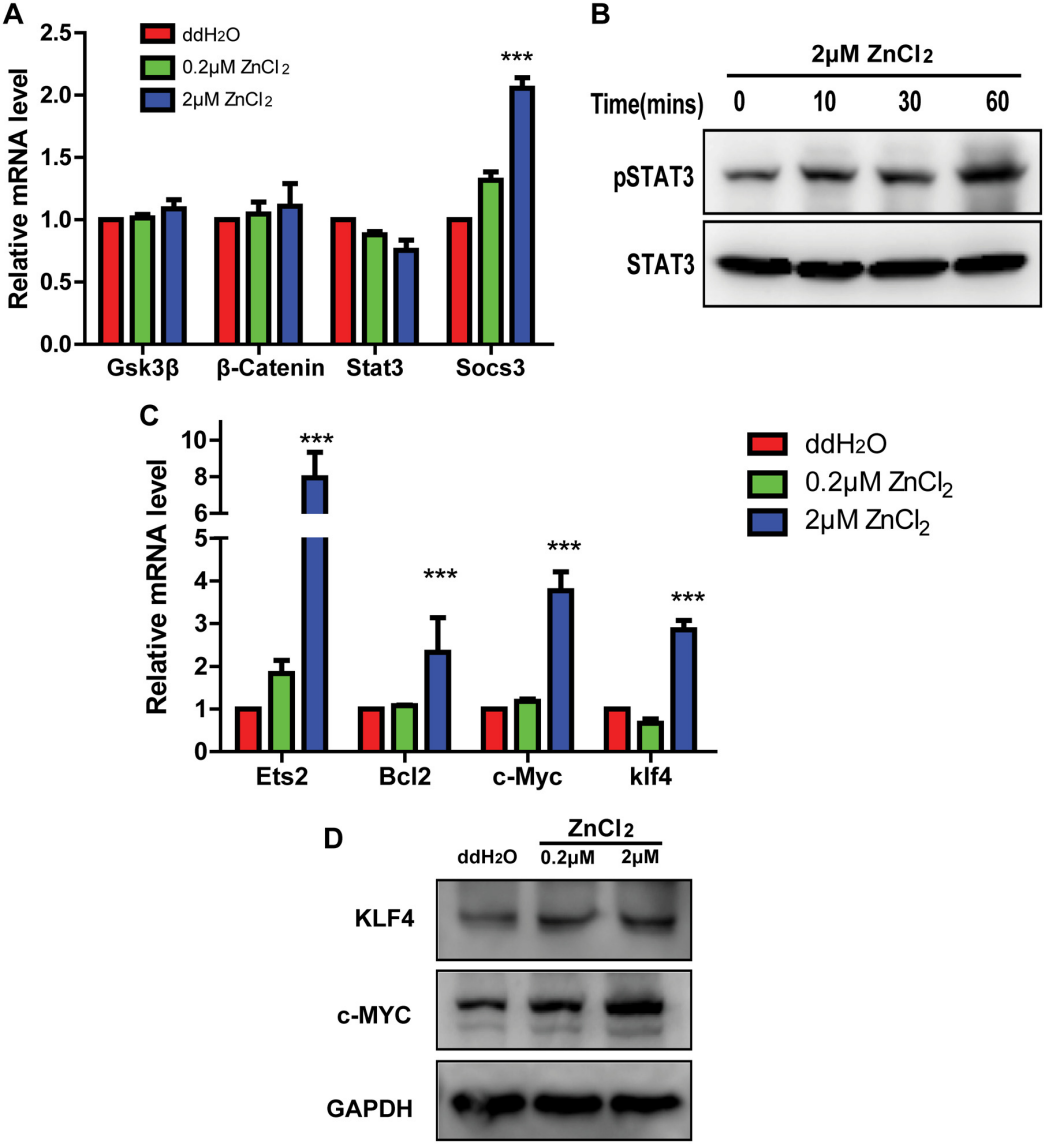
Zinc activates Stat3 signaling. All cells were cultured in LIF withdrawal medium. (A): qRT-PCR analyses of the expression levels of genes belong to Wnt signaling pathway (Gsk3β, β-Catenin) and Stat3 signaling pathway (Stat3, Socs3) in cells treated with ddH_2_O and ZnCl_2_ (0.2μM and 2μM) for 48 hours. The data are displayed relative to the results of the ddH_2_O treatment group and represented as mean±SEM; n = 3. *** indicates p<0.001. (B): Western blot analyses of the expression of STAT3 and pSTAT3 in cells treated with 2μM ZnCl_2_ for 0, 10, 30 and 60 minutes. (C): qRT-PCR analyses of the expression of Stat3 targeting genes in cells treated with ddH_2_O and ZnCl_2_ (0.2μM and 2μM) for 48 hours. The data are displayed relative to the results of the ddH_2_O treatment group and represented as mean±SEM; n = 3. *** indicates p<0.001. (D): Western blot analyses of the expression of Stat3 targeting genes (c-Myc and Klf4) in cells treated with ddH_2_O and ZnCl_2_ (0.2μM and 2μM) for 48 hours.

The pluripotency and self-renewal maintenance of ES cells requires a broad cell signaling network, and numerous studies showed that LIF/Stat3 signaling pathway is indispensable for the pluripotential phenotype of mouse ES cells [12–15]. A previous study demonstrated that zinc regulated inflammatory response through Jak/Stat3 pathway [33]. According to our results presented above that ZnCl_2_ can promote mouse ES cell pluripotency phenotype in the absence of LIF, we next examined whether ZnCl_2_ exerted its pro-pluripotency function on mouse ES cells through regulating Jak/Stat3 signaling pathway. Wnt and Stat3 signaling pathways are very important for mouse ES cell fate determination [34]. Therefore, we detected the expression levels of genes belong to Wnt pathway (Gsk3β, β-Catenin) and Stat3 pathway (Stat3, Socs3) via qRT-PCR. After 48 hours of 2μM ZnCl_2_ treatment, the expression levels of Gsk3β, β-Catenin and Stat3 remained unchanged (Fig 5A), but the expression of Stat3 target gene (Socs3) increased significantly (Fig 5A), suggesting that Stat3 signaling was upregulated, whereas Wnt signaling was not changed following ZnCl_2_ treatment. We then detected the posttranslational modifications of key proteins in Stat3 pathway. Stat3 is active when Tyr705 is phosphorylated. We incubated the cells with 2μM ZnCl_2_ for 0, 10, 30 and 60 minutes in LIF withdrawal medium and examined the protein levels of Stat3 and phosphorylated Stat3 (pTyr705) via western blotting. It showed that 2μM ZnCl_2_ treatment for 60 minutes elevated pSTAT3 level significantly (Fig 5B). We further analyzed the expression levels of Stat3 target genes via qRT-PCR and western blotting. After 48 hours 2μM ZnCl_2_ treatment, the expression levels of Ets2, Bcl2, c-Myc and Klf4 were all increased significantly (Fig 5C and 5D). Next, we prolonged the incubation time and then analyzed the effect of ZnCl_2_ on Stat3 signaling. Compared with 2i treated cells, the protein levels of pSTAT3 decreased gradually after 2μM ZnCl_2_ treatment for 72 hours,96 hours and 120 hours (S2A Fig). The mRNA levels of Stat3 target genes, Klf4 and Socs3, also decreased gradually as the incubation time was prolonged (S2B Fig). Collectively, these results suggested that zinc transiently activated Stat3 through phosphorylation and played a particularly important role in the transcriptional network controlling pluripotency of mouse ES cells.

### Inhibition of Stat3 signaling abrogates the effect of ZnCl_2_ on mouse ES cell pluripotency

**Fig 6:**
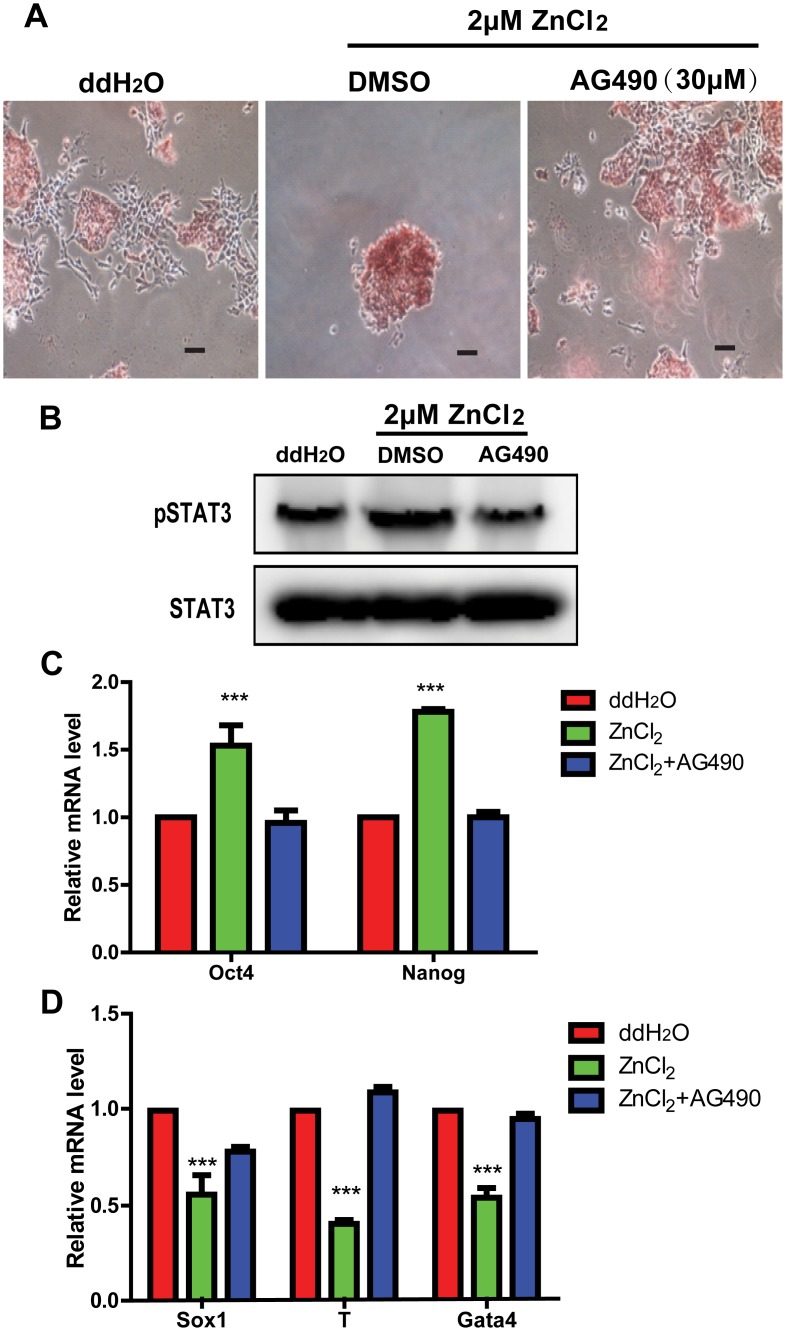
Inhibition of Stat3 signaling abrogates the effect of ZnCl_2_ on mouse ES cell pluripotency. All cells were cultured in LIF withdrawal medium and divided into three groups: cells treated with ddH_2_O, cells treated with 2μM ZnCl_2_ + DMSO for 48 hours and cells treated with 2μM ZnCl_2_ + AG490 (30μM) for 48 hours. (A): Alkaline phosphatase staining of three group cells. Bars = 40μm. (B): Western blot analyses of the expression of STAT3 and pSTAT3 in three group cells. (C): qRT-PCR analyses of the expression of Oct4 and Nanog in three group cells. The data are displayed relative to the results of the ddH_2_O treatment group and represented as mean±SEM; n = 3. *** indicates p<0.001. (D): qRT-PCR analyses of the expression of lineage-specific markers in three group cells. The data are displayed relative to the results of the ddH_2_O treatment group and represented as mean±SEM; n = 3. *** indicates p<0.001.

Based on the findings that Stat3 activity and its target genes were altered after ZnCl_2_ treatment, we hypothesized that Stat3 signaling pathway might be associated with the effect of ZnCl_2_ in promoting the pluripotent potential of mouse ES cells. Jak is an upstream factor in Jak/Stat3 pathway, which can activate Stat3. AG490 is a selective Jak inhibitor that inhibits Jak/Stat3 signaling [35]. We thus treated mouse ES cells with AG490 (30μM) for 48 hours in LIF withdrawal medium. Compared with the ddH_2_O treatment, 2μM ZnCl_2_ treatment promoted ES cells clone morphology and increased AP enzyme activity. However, when AG490 was added into medium, the cells regained their differentiated morphology (Fig 6A). AG490 treatment also decreased the protein level of phosphorylated STAT3 (pTyr705) (Fig 6B) as well as the mRNA levels of Oct4 and Nanog (Fig 6C) in 2μM ZnCl_2_ treated cells. Furthermore, compared with only ZnCl_2_ treated cells, the expression of differentiation genes increased in ZnCl_2_ plus AG490 treated cells (Fig 6D). Collectively, these findings indicated that zinc regulated the mouse ES cell pluripotency potential mainly through Stat3 signaling.

## Discussion

Small molecules are useful chemical tools for manipulating cell fate, and clearly offer some distinct advantages over genetic manipulation. For example, small molecules are nonimmunogenic, more cost-effective, more easily synthesized, preserved, and standardized. Their effects are often reversible and can be finely tuned by varying the concentrations. Previous studies have shown that many small molecule compounds play essential roles in maintaining ES cell self-renewal, pluripotency and reprogramming [10, 11, 36, 37]. However, to date, the chemical regulation of ES cell fate remains to be further investigated to meet the needs of regenerative medicine. We found that, compared with ddH_2_O treated cells, mouse ES cells maintained the clone morphology and a stronger AP enzyme activity under ZnCl_2_ exposure for 48 hours at a final concentration of 2μM in LIF withdrawal medium (Fig 1A and 1B). ZnCl_2_ treatment also increased the expression of pluripotency markers and inhibited the expression of differentiation genes (Fig 1C and 1D). Importantly, we observed similar phenomenons in RA and EB differentiation assays (Fig 2). Our study indicates that ZnCl_2_ might significantly promote pluripotency of mouse ES cells. However, Corradi et al. reported that the EB formation and cardiac differentiation of mouse ES cells were inhibited by zinc oxide (ZnO) nanomaterials at noncytotoxic doses [38]. It is possible that some zinc ions are reacted to form ZnO in mouse ES cell culture medium in our study. Therefore, ZnO may also participate in the maintenance of mouse ES cell pluripotency. We also observed that the mouse ES cells started the trend of differentiation after ZnCl_2_ treatment for 72 hours, 96 hours and 120 hours (Fig 3). Mouse ES cells can be maintained indefinitely in the pluripotent state in LIF plus serum medium *in vitro*. By contrast, pluripotency is a transitory state that exists only during a short window of early embryo development *in vivo*. Pluripotency is also a highly regulated state *in vivo*. However, the regulatory mechanism is very complex which has not been well elucidated now. Zinc, which is an essential trace element for mammals, could transiently maintained mouse ES cell pluripotency *in vitro* in our study. Therefore, we speculate that zinc may be an important regulator for the pluripotency maintenance of early embryo development. These all need to be elucidated in future studies.

Similarly, there are studies demonstrating that other ions are also critically involved in ES cell biology. Embryos depend on a ubiquitous, persistent and highly versatile signaling system that is based around a single messenger, Ca^2+^ [39]. Another study showed that iron (Fe^3+^) is essential for porcine embryonic development [40]. Therefore, our data lend support to the notion that these important metal elements are essential regulators for embryonic pluripotency and development.

Stat3 is tightly integrated into the gene regulatory network of pluripotency. Chen and Kidder et al. found that Stat3 bound to downstream gene promoters for gene activation, including Klf4 and c-Myc. They also observed that Stat3 co-occupied pluripotency gene promoters with Oct4, Sox2 and Nanog [34, 41]. Although Stat3 target genes have been extensively identified, little is known about its upstream chemical regulators, especially its natural chemical regulator. ZnCl_2_ is an indispensible natural small molecule for mammal. Here, we found that ZnCl_2_ at appropriate concentration promoted Stat3 activities. Although ZnCl_2_ did not enhance Stat3 expression, it activated Stat3 through phosphorylating Tyr705. Then, activated Stat3 promoted target genes expression to maintain pluripotency. Stat3 and its target genes such as Klf4 and c-Myc are essential for maintaining mouse ES cell self-renewal and pluripotency [28, 41]. In our study, we have provided significant data to support that ZnCl_2_ promote mouse ES cells pluripotency, in part, through activating Stat3. However, it remains possible that ZnCl_2_ exerts its effects on mouse ES cell pluripotency through activating or inhibiting other factors besides Stat3. This needs to be elucidated in future studies.

Although human and mouse ES cells are differed in the signaling networks and epigenetic landscapes, it has been revealed that they share the same core regulators Oct4/Sox2/Nanog and similar transcriptional regulatory network [42]. Given that zinc is an important trace element for both human and mouse and zinc promoted the expression of Oct4, Sox2 and Nanog in our study (Figs 1C, 1D and 2B–2E), we propose that zinc is extremely possible to have similar functions regarding the maintenance and acquaintance of pluripotency in human cells, which is very worthy to be elucidated in future studies.

**Fig 7:**
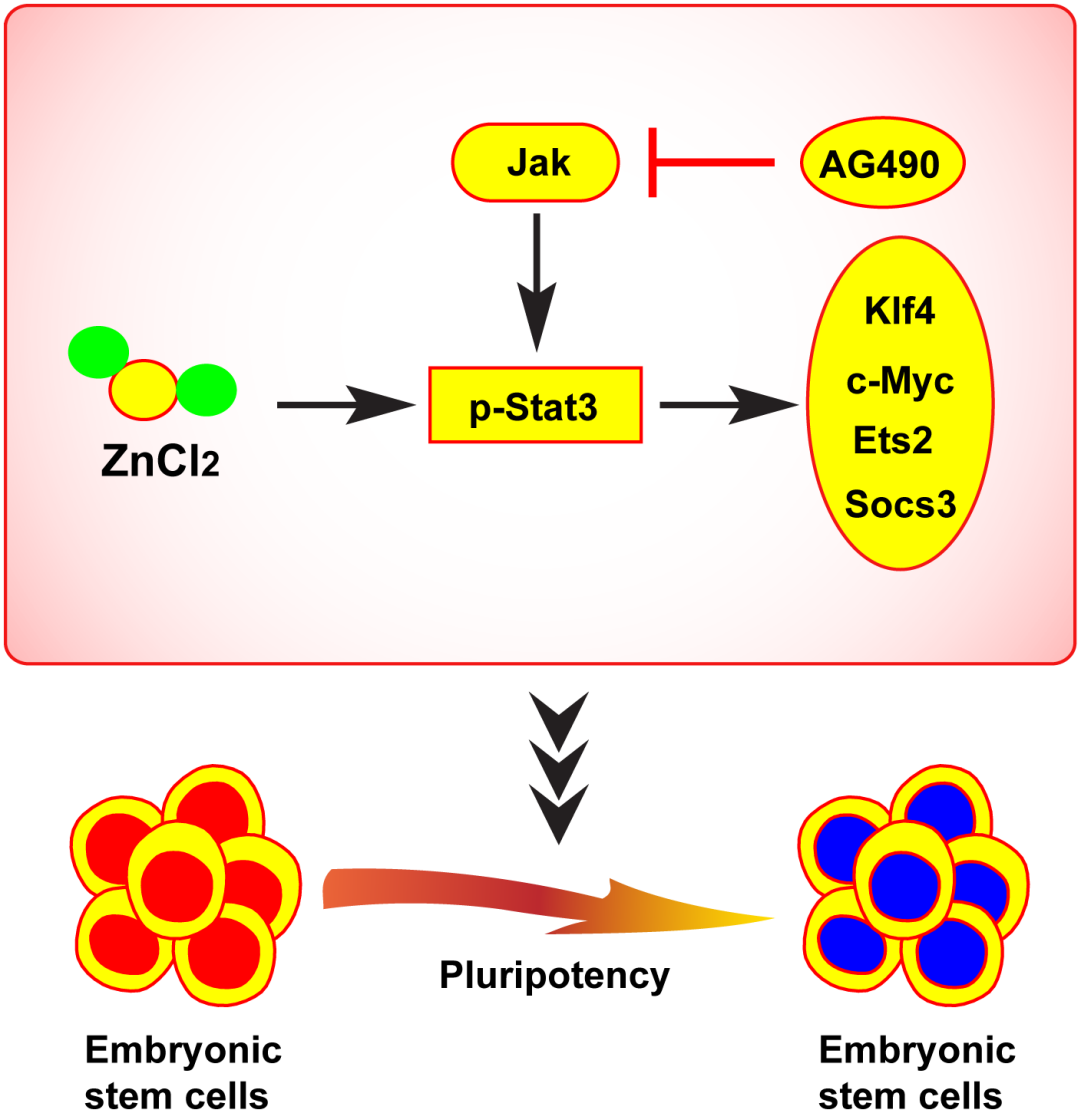
Schematic diagram of the regulatory role of ZnCl_2_ in the pluripotency maintenance of ES cells.

In summary, we have clearly demonstrated for the first time that ZnCl_2_ promoted mouse ES cell pluripotency in differentiation conditions through activating Stat3 pathway. Briefly, ZnCl_2_ stimulation activates Stat3 through phosphorylating. Then, activated Stat3 increases its target genes expression to promote embryonic stem cell pluripotency (Fig 7). Our finding not only has implications for zinc’s role in ES cell biology, but also elucidates another potential control switch for ES cell fate in regenerative medicine. Given the current challenges of pluripotency maintenance, this research and the questions raised by this work demonstrate the need for continued studies into the molecular basis for pluripotency and the role of zinc in mouse ES cells, as well as its relevance to human embryonic stem cells.

## Supporting Information

S1 Fig Zinc does not affect mouse ES cell proliferation in the presence of LIF. All cells were cultured in medium supplemented with LIF. (A): Immunostaining images of cells treated with ddH2O and ZnCl2 (0.2μM and 2μM) for 48 hours. Cells were stained with antibody against Ki67. Nuclei were counterstained with Hoechst33342. Bars = 40μm. (B): Growth curves of cells treated with ddH2O and ZnCl2 (0.2μM and 2μM) were constructed using the CCK-8 assay. The data are represented as mean±SEM; n = 3.
(TIF)

S2 Fig The alteration of Stat3 signaling on ZnCl_2_ treatment for different times. (A): Western blot analyses of the expression of STAT3 and pSTAT3 in cells treated with 2μM ZnCl_2_ or 2i for 24, 48, 72, 96 and 120 hours. (B): qRT-PCR analyses of the expression levels of Stat3 target genes, Klf4 and Socs3, in cells treated with 2μM ZnCl_2_ or 2i for 24, 48, 72, 96 and 120 hours. The data are displayed relative to the results of the 2i treatment for 24 hours group and represented as mean±SEM; n = 3.
(TIF)

S1 Table qRT-PCR primers. All primers used for detecting mRNA expression levels by qRT-PCR.
(DOC)
