# Supplementary figures and images for: Zinc Chloride Transiently Maintains Mouse Embryonic Stem Cell Pluripotency by Activating Stat3 Signaling
Source: PLoS One. 2016 Feb 24;11(2):e0148994. doi: 10.1371/journal.pone.0148994 (PMC4765890; doi:10.1371/journal.pone.0148994)

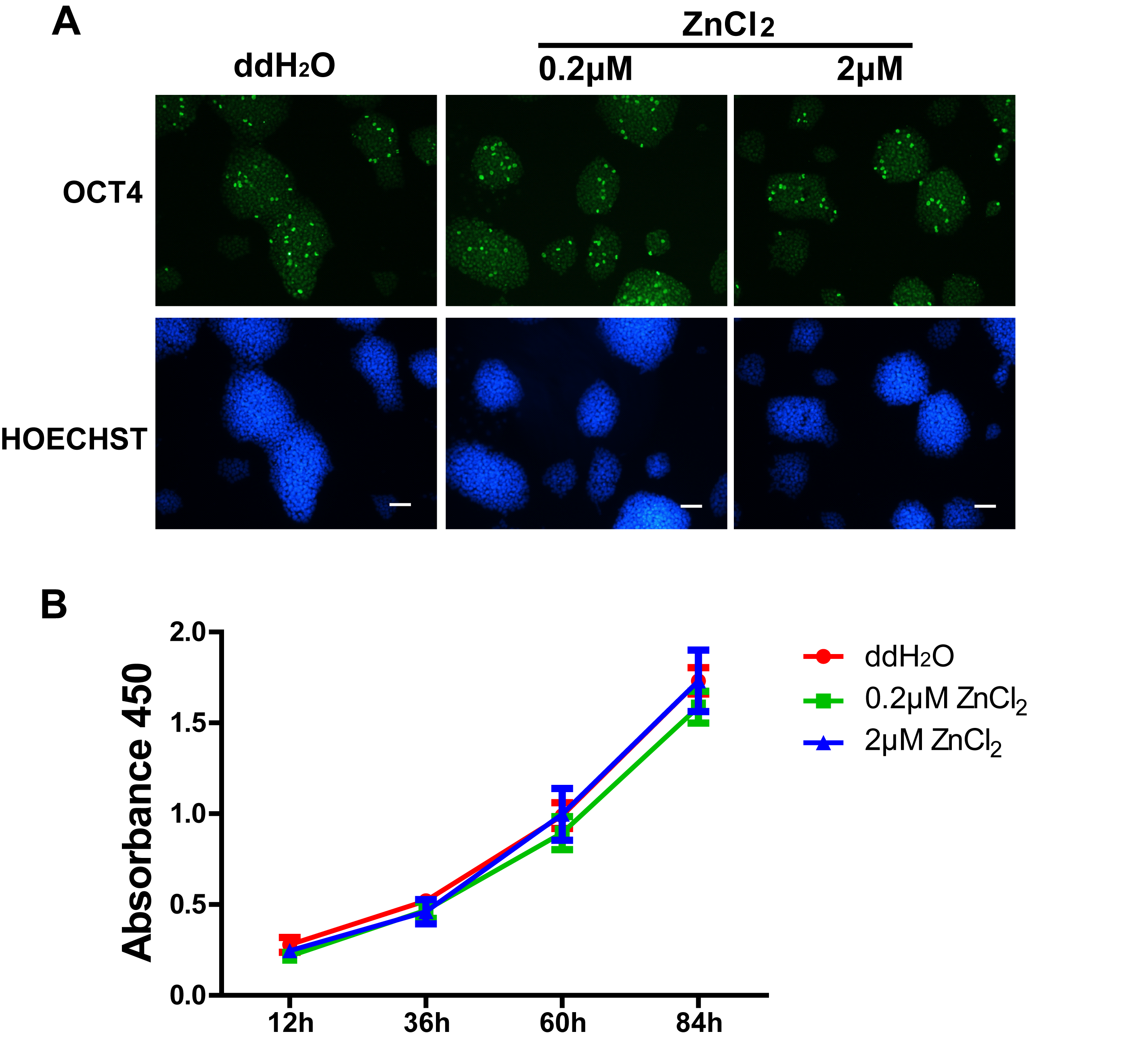

Supplement: S1 Fig — All cells were cultured in medium supplemented with LIF. (A): Immunostaining images of cells treated with ddH2O and ZnCl2 (0.2μM and 2μM) for 48 hours. Cells were stained with antibody against Ki67. Nuclei were counterstained with Hoechst33342. Bars = 40μm. (B): Growth curves of cells treated with ddH2O and ZnCl2 (0.2μM and 2μM) were constructed using the CCK-8 assay. The data are represented as mean±SEM; n = 3. (TIF) [file pone.0148994.s001.tif]

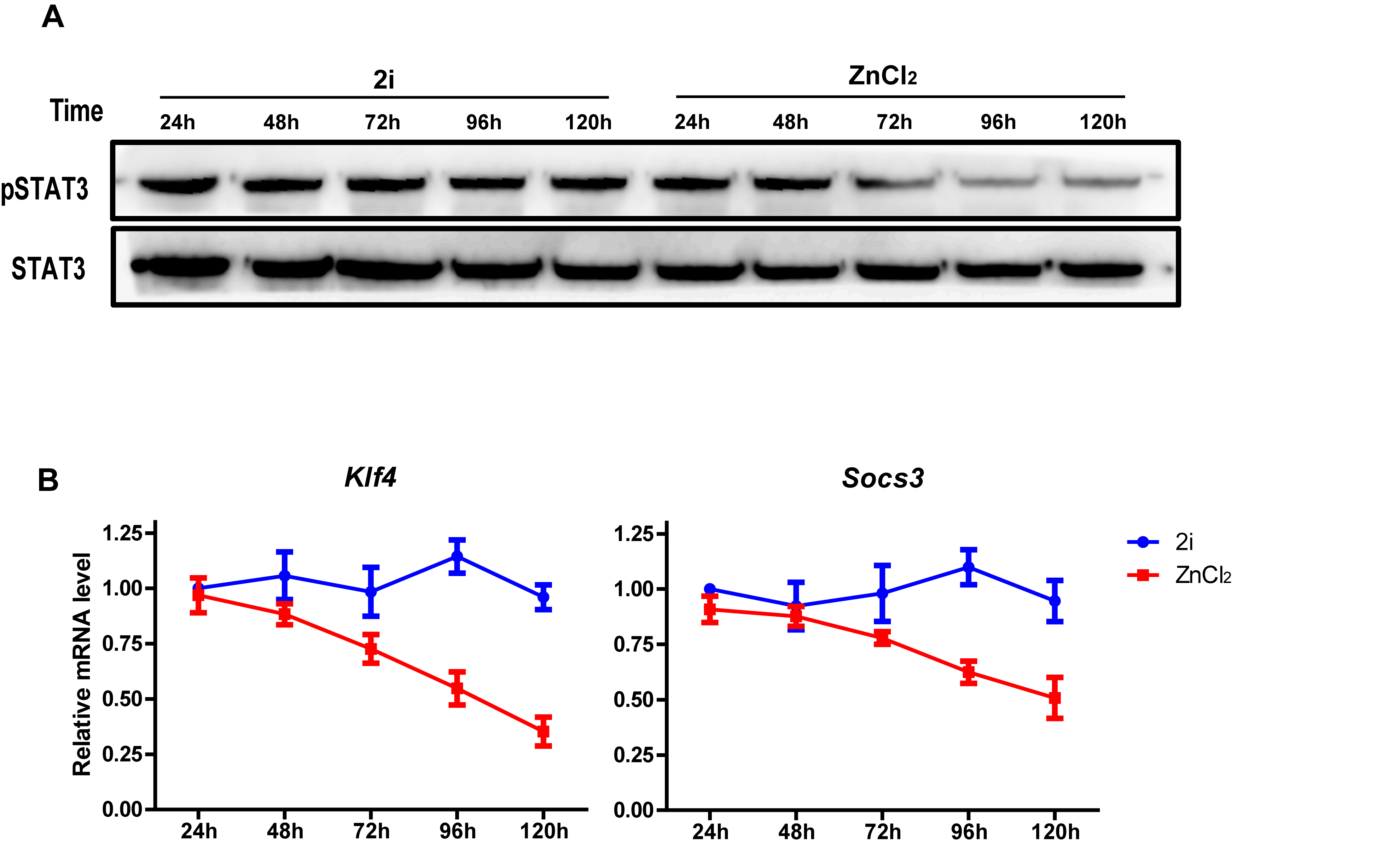

Supplement: S2 Fig — (A): Western blot analyses of the expression of STAT3 and pSTAT3 in cells treated with 2μM ZnCl2 or 2i for 24, 48, 72, 96 and 120 hours. (B): qRT-PCR analyses of the expression levels of Stat3 target genes, Klf4 and Socs3, in cells treated with 2μM ZnCl2 or 2i for 24, 48, 72, 96 and 120 hours. The data are displayed relative to the results of the 2i treatment for 24 hours group and represented as mean±SEM; n = 3. (TIF) [file pone.0148994.s002.tif]
