# Supplementary material for: Zinc Chloride Transiently Maintains Mouse Embryonic Stem Cell Pluripotency by Activating Stat3 Signaling
Source: PLoS One. 2016 Feb 24;11(2):e0148994. doi: 10.1371/journal.pone.0148994 (PMC4765890; doi:10.1371/journal.pone.0148994)
Supplement: S1 Table — All primers used for detecting mRNA expression levels by qRT-PCR. (DOC) [file pone.0148994.s003.doc]

**S1 Table. qRT-PCR primers**

| Name | Sequence |
| --- | --- |
| Oct4 PF | TCTTTCCACCAGGCCCCCGGCTC |
| Oct4 PR | TGCGGGCGGACATGGGGAGATCC |
| Sox2 PF | TAGAGATAGACTCCGGGCGATGA |
| Sox2 PR | TTGCCTTAAACAAGACCACGAAA |
| Nanog PF | GCTCAGCACCAGTGGAGTATCC |
| Nanog PR | TCCAGATGCGTTCACCAGATAG |
| Sox1 PF | AAGGAACACCCGGATTACAAGT |
| Sox1 PR | GTTAGCCCAGCCGTTGACAT |
| T PF | GGTGGCTTGTTCCTGGTGC |
| T PR | GTAGGTGGGCTGGCGTTAT |
| Gata4 PF | CCTGGAAGACACCCCAATCTC |
| Gata4 PR | AGGTAGTGTCCCGTCCCATCT |
| Gsk3β PF | GTCCGACTGCGGTATTTCTTC |
| Gsk3β PR | CTCGATGGCAGATTCCAAAGG |
| β-Catenin PF | TCCCATCCACGCAGTTTGAC |
| β-Catenin PR | TCCTCATCGTTTAGCAGTTTTGT |
| Stat3 PF | AGAACCTCCAGGACGACTTTG |
| Stat3 PR | TCACAATGCTTCTCCGCATCT |
| Socs3 PF | ATGGTCACCCACAGCAAGTTT |
| Socs3 PR | TCCAGTAGAATCCGCTCTCCT |
| c-Myc PF | TGACCTAACTCGAGGAGGAGCTGGAATC |
| c-Myc PR | AAGTTTGAGGCAGTTAAAATTATGGCTGAAG |
| Bcl2 PF | GTCGCTACCGTCGTGACTTC |
| Bcl2 PR | CAGACATGCACCTACCCAGC |
| Klf4 PF | GTGCCCCGACTAACCGTTG |
| Klf4 PR | GTCGTTGAACTCCTCGGTCT |
| Ets2 PF | CCTGTCGCCAACAGTTTTCG |
| Ets2 PR | TGGAGTGTCTGATCTTCACTGA |
| Gapdh PF | TGCACCACCAACTGCTTAGC |
| Gapdh PR | GGCATGGACTGTGGTCATGAG |
